# Professional Secrecy

**Published:** 1857-11

**Authors:** 


From the Medical Chronicle of Montreal.

### *Professional Secrecy.*

The relations of a physician to those who favor him with their confidence are of so intimate a nature; he has so many opportunities to become acquainted with matters, occurring in families, that ought to be held sacred by him and remain securely locked in the deepest recesses of his bosom; he is so often selected as the confidant to whom the wretched, sorrowful, and repentant intrust their tale of guilt, remorse, and grief, it becomes him to be a man of extended sympathies and high uncompromisable honor to command the esteem of his patients, and make them feel that their simplest as well as greatest secrets are perfectly safe in his keeping. From the earliest periods in the history of medicine down to recent times, the fathers in medicine, and those intrusted with the preparation of the medical neophyte for the practice of the important duties of his professsion, have thought it incumbent on them to exact an oath from each successful candidate, that he would never betray any secret intrusted to his keeping, or one that he should accidently become cognizant of, professionally, ere they invested him with the authority to go forth into the world and assume the responsibilities of physician to his fellows. It was never intended, however, that a medical man was to remain silent whenever facts relating to fearful crime, such as murder, either accomplished or contemplated, came to his knowledge. No oath could be binding on a man, or warrant him in assisting to defeat the ends of justice. Under such circumstances silence would be criminal, and make him accessary to the fact. Instead of meriting praise for keeping secrets of that nature, he would rather deserve the execrations of society.

An event has lately occurred in the city of New York which has caused a great deal of excitement in the community, and involves the question of the betrayal of trust on the part of a physician. We propose laying, in brief terms, the facts of the case before our readers, with our views on the subject. The notorious Mrs. Cunningham, *alias* Mrs. Burdell, who a short time since was tried for the murder of Dr. Harvey Burdell, and acquitted, has lately attempted the perpetration of a criminal fraud, formed for the purpose of obtaining the whole of the late Dr. Burdell's property. It appears that while this lady was confined in the Tombs awaiting her trial for murder, she caused it to be announced that she was pregnant with child, and that in due time an heir would be born to Dr. Burdell's estate. Her personal appearance justified the assertion, and, at her request, Judge Dean gave an official notification of the circumstance. Mrs. C. next consulted Dr. Uhl, who would appear to have been her medical adviser, and desired to engage his services. Subsequently she told him that her pregnancy was a pretence, but that if he would aid her in carrying out her plans to a successful issue, one thousand dollars would be his fee. The services of one Dr. Batlin, an individual whom Mrs. Cunningham professes "to have in her power," were also secured for the interesting occasion. Dr. Uhl apparently assented, but his conscience not feeling very easy, he immediately informed District Attorney Hall of everything that had transpired. By the advice and at the urgent request of Mr. Hall, Dr. Uhl entered enthusiastically into Mrs. C.'s plans, with the view of exposing the attempted fraud and bringing the guilty ones to justice. "To carry on the project, he represented that he had the good luck to have found a woman who was about being confined in Elm Street, who would part with her baby. She was neither to see or know Mrs. Cunningham, and therefore there could be no possibility of any unpleasant developements. Apartments were procured at 190 Elm Street, and were furnished by Mr. Hall for the proper reception of a lay-ing-in woman. Meantime officers Dilks, Hopkins, Speight, and Walsh were detached to keep a close look-out in Bond Street, Mr. Hall assisted himself on Monday in perfecting arrangements. An infant that had been born on Saturday was engaged from its mother at Bellevue Hospital, marked by lunar caustic, and otherwise identified, and a nurse engaged. Dr. Uhl was to be at Elm Street to deliver over the baby to the Sister of Charity who was to call for it.

All things being ready, Mrs. Cunningham was notified that the heir was born, and she said she would send a lady (whose name she refused to give) to look at the place, so as to know where to go at a later hour. Dr Uhl stationed himself at 190 Elm Street, and kept watch for the lady who was to be sent to reconnoitre the premises; and in a short time a person made her appearance, passed by and inspected the place. That lady, although in the disguise of Charity, Dr. Uhl recognized as Mrs. Cunningham herself. As soon as she had left the neighborhood, Dr. Uhl again visited No. 31 Bond Street, when Mrs. Cunningham called a lady, whom Dr. Uhl recognized as her sister, (Mrs. Burns,) into the room, and asked her if she was ready to go for the child, when Mrs. Burns asked for the dark dress, and Mrs. Cunningham told her where it was. It was then arranged by Mrs. C. and the Doctor that he should go to No. 190 Elm Street, and wait at the front hall till the lady to be sent should come; and in order to avoid any mistake in the matter, the lady was to carry a white handkerchief in her hand. After waiting about fifteen minutes, the lady appeared who had previously reconnoitred the premises, carrying, as agreed upon, a white handkerchief in her hand. The lady wore a long black dress, and a hood or close bonnet, after the style worn by the Sisters of Charity—her face being almost covered; but from her manner, form, and general bearing, Dr. Uhl again recognized her to be none other than Mrs. Cunningham herself. Dr. Uhl asked her if she had come for the child. She made no reply, but followed him up stairs to the door of the room. The light burnt dimly on the centre table, the door which opened into the adjoining room displayed the foot of a cot on which the sick mother was supposed to be prostrate. Mrs. Cunningham only looked in, but a glance must have satisfied her all was right; the nurse, Mary Regan, sat with the child in her lap, the basket at her feet; as Mrs. Cunningham presented herself, she was asked if she came for the child; as agreed upon, she shook her handkerchief in reply; the next instant the "little thing" was placed in the basket and handed through the partially opened door, and Mrs. C. hurriedly left the house. Dr. Uhl then started for home leaving those engaged with him in the plot to attend to Mrs. Cunningham's movements. The doctor had been at home but a brief period, when he received a summons through a gentleman (a stranger) to repair immediately to 31 Bond Street, as Mrs. Burdell was then suffering with labor pains. On arriving at the residence of Mrs. Cunningham, he was conducted to a darkened room, where Mrs. Cunningham was in bed and apparently in great suffering. Dr. Catlin and Mrs. Burns, sister to Mrs. Cunningham, were present. Dr. Catlin brought in a pail containing blood, with which the sheets were saturated; and, in due time, after considerable groaning and moaning, the expectant heir was brought forth and transferred over to the nurse, Jane Bell, who washed and dressed it, while the doctors went through the process of bandaging the suffering,bu' delighted mother, who took occasion to exclaim, with much earnestness, that "she had put her trust in God, and in return he had been pleased to favor her!" At this stage, Dr. Uhl left the house and the case to the charge of others, who were on hand at the door. All this farce was performed on Monday night, between eleven and twelve o'clock. At half past eight the policemen in the secret took their stations, Capt. Dilks in Broadway opposite Bond Street, Capt. Speight opposite Burdell's house. Capt. Hopkins took up his station in the alley which leads from the rear of 31 Bond into Bleckei Street, when he was mistaken for a burglar, much to the alarm of the neighborhood. Capt. Speight saw Mrs. Cunningham come out of the house, followed her to Elm Street, and saw her return to Bond Street, the basket containing the baby in her possession. The policemen then took their stations in front of the house to observe who went in or out. Among the latter was Catharine Bell, the nurse, Dr. Catlin, and lastly Dr. Uhl. About twelve o'clock at night the police started towards Broadway, when they met District Attorney Hall, Capt. Dilks, Dr. Montagnie, and officers Smith, Wilson, and Walsh.

After a little conversation, it was agreed that Capt. Dilks and Dr. Montagnie should go to 31 Bond Street, and state that they had heard that a curious delivery had taken place, and that they wanted to see that all was right. Two women answered the summons, and stated that Mrs. Cunningham was too sick to be seen. The women then went up stairs, and the men followed. Before reaching what was Dr. Burdell's bedroom, the women opened the door and said, "Mrs. Burdell, here are two gentlemen who want to see you." She said, "Shut the door; they can't come in." Dilks immediately went in and said, "Madam, we don't wish to interrupt you seriously, but we have heard that you have been delivered under suspicious circumstances, and it is our duty to inquire." The light was then down; they turned it up, and saw by her side a sleeping infant. Dr. Montagnie recognized it as the child he had carried to Elm Street. He had previously marked it with a little lunar caustic under each armpit and under each ear, marks which did not appear until next day. He had also cut the umbilical cord anew, and retied it with the edging of a pocket-handkerchief which there could be no mistaking.

Dr. Montagnie said to Mis. Cunningham, "Whose child is this?" She said, "It is my child." He asked if it was the child of Dr. Burdell.

She said, " Yes, of course ! whose else could it be ? I am his widow."

Almost immediately Dilks came down to the door, and the police went up stairs. Those present were apprehensive that the child might be killed, and an attempt was made to take it away from her at all hazards. She said, " Don't take my baby !" and the woman persisted in saying, " You must not take this baby ; it is Mrs. Burdell's baby." One of the police asked, " Where is the basket that it was brought here in ?" She said, " There is no basket in the place. A policeman then said, " There was no use making any disturbance about it ; the doctors were arrested, and everything was found out ; the child belonged to the Bellevue Hospital. The hospital clothes had been taken from it, and new and elegant apparel, evidently made for the purpose, had been put upon it. In the back room—the room in which Dr. Burdell was murdered—the police found the remains of a lunch. Meanwhile Mrs. Cunningham still persisted that she had been in labor, and was suffering with afterpains. The basket could not be found, high or low. The after-birth that Dr. Montagnie brought from the Bellevue Hospital was there ; a pail of bullock's blood was found, and the sheets smeared with blood."

Now, the question which has been mooted in the United States, in reference to the conduct of Dr. Uhl, viz : Was he warranted in betraying secrets revealed to him professionally ? is, we conceive, easily answered. Mrs. Cunningham contemplated the commission of a crime which would, if successfully carried out, have seriously injured the interests of other parties. It was absolutely necessary to the success of the plot that a physician should be made cognizant of the whole details, and induced to work, in harmony with others. Whatever guilt was attachable to any one concerned in the criminal act would be equally attachable to him. When Mrs. Cunningham, therefore, informed Dr. Uhl of the fraud which she intended to perpetrate, and requested him to become a party to it by an attendance on her during the pretended accouchment, she virtually asked him to criminate himself by aiding her to play the villain. The oath which Dr. Uhl subscribed to on the day of his graduation was never intended to impose silence on him under such circumstances. His duty to himself, to society, and to the miserable woman was perfectly clear. When the proposition was made, he ought immediately to have declined having anything to do with it ; he should have given her distinctly to understand that he would not consider himself bound to withhold his evidence against her in the event of the crime being committed ; and lastly, he should have seriously warned her against the criminal course she intended to pursue. In neglecting to act in this manner, pursuing, indeed, a most opposite course, and *not* in betraying professional secrets, has Dr. Uhl, in our opinion, acted unprofessionally, and laid himself open to the severest censures of his confreres. Instead of firmly and manfully refusing on the instant to have lot or part in the fraud, he willfully deceives Mrs. Cunningham by accepting with apparent eagerness the terms of her proposition. He not only does this, but he informs District Attorney Hall of what has transpired, at whose request, moreover, he consents to act out a falsehood, and by so doing lead on a female to the commission of a crime. We have yet to learn that such an act is either morally right, or one that is becoming in a member either of the legal or medical profession. It is the undoubted duty of all to prevent the commission of crime, and to aid in its detection when committed; but who ever tempts his fellow to do a criminal action by placing facilities in his way, ought certainly to be considered an accessory, and judged accordingly. Dr. Uhl has acted unprofessionally also in becoming the agent of Mr. Hall, a sort of detective or spy in the service of that gentleman. Had he, when he lodged information with the authorities, washed his hands of all further connection with the deceit, we cold have nothing to say against him; but by becoming the very life and soul of every move in the deception, he prostituted the noble profession to which he belongs, and should, as a punishment, be deprived of the status he holds in that profession.

